# Prevalence and distribution of *Taenia solium* cysticercosis in naturally infected pigs in Punjab, India

**DOI:** 10.1371/journal.pntd.0006960

**Published:** 2018-11-15

**Authors:** Satinder Pal Singh, Balbir Bagicha Singh, Deepali G. Kalambhe, Devendra Pathak, Rabinder Singh Aulakh, Navneet K. Dhand

**Affiliations:** 1 School of Public Health & Zoonoses, Guru Angad Dev Veterinary and Animal Sciences University, Ludhiana, Punjab, India; 2 Department of Veterinary Anatomy, Guru Angad Dev Veterinary and Animal Sciences University, Ludhiana, Punjab, India; 3 Sydney School of Veterinary Science, The University of Sydney, Camden, NSW, Australia; Universidad Peruana Cayetano Heredia, PERU

## Abstract

**Background:**

*Taenia solium* (*T*. *solium*) cysticercosis remains a neglected zoonotic disease in India. The current study was planned to estimate the prevalence of *T*. *solium* porcine cysticercosis in the Punjab state of India, to compare this prevalence with the disease prevalence in pigs reared outside Punjab and to assess the distribution of the parasite in pig carcasses.

**Methods:**

Two slaughter shops were selected in each of the 22 districts of Punjab. Pigs slaughtered on the day/s of inspection were post-mortem inspected to identify the presence of *T*. *solium* cysts. Estimated true prevalence was estimated by taking into account the diagnostic sensitivity (38%) and specificity (100%) of post-mortem inspection using the Rogan-Gladen estimator. Positive carcasses were purchased and brought to the laboratory to assess the tissue distribution of *T*. *solium* cysts and to conduct PCR targeting large subunit rRNA gene, internal transcribed spacer 1 gene, ITS1 gene and Cytochrome oxidase I gene. The selected PCR products were submitted for sequencing and phylogenetic analyses were performed.

**Findings:**

We contacted 71 shop owners to achieve a sample of 44 shops for the study. We inspected 642 pigs reared in Punjab and 450 imported from other states at these slaughter shops. In addition, we sampled 40 pigs from an abattoir located in the state capital. Of the 642 pigs reared in Punjab, 9 had *T*. *solium* cysts with an apparent prevalence of 1·40% (95% CI: 0·74%, 2·64%) and the estimated true prevalence of 3.69% (95% CI: 1·95%, 6·95%). Pigs imported from outside the state had a significantly higher prevalence (odds ratio: 2·58; 95% CI: 1·12, 5·98; p-value: 0·026) as 15 of the 450 imported pigs were positive (apparent prevalence: 3.33%; 95% CI: 2.03%, 5.43%; estimated true prevalence: 8.77%; 95% CI: 5.34%, 14.28%). None of samples was positive from the pigs sampled at the abattoir in the state capital. The PCR confirmed *T*. *solium* cysts from all the 24 positive samples. We counted a median of 897 (range 526–1964) cysts per infected pig from the 19 infected pig carcasses inspected. The phylogenetic tree based on the alignment of partial cytochrome oxidase 1 sequences indicated all positive samples to be clustered with the *T*. *solium* Asian genotype. The analysis did not indicate the presence of *T*. *asiatica* in the slaughter pigs.

**Conclusions:**

Despite the underestimation of the prevalence due to missing mildly-infected carcasses, low participation and lack of representative sampling, the presence of heavily infected carcasses containing viable cysts, particularly those imported from outside the state, indicates that *T*. *solium* cysticercosis is an important food safety concern for pork consumers in Punjab, India. Measures should be taken to reduce the disease prevalence in pigs to reduce the disease burden in the public.

## Author summary

*Taenia solium* cysticercosis is a neglected zoonosis and severely affects pork production and public health in India. The current study was conducted to estimate the prevalence and distribution of *T*. *solium* porcine cysticercosis in the Punjab state of India and to compare the disease prevalence in pigs reared within and outside Punjab. Overall, 24 of the 1132 inspected pigs had viable cysts. Imported pigs had significantly higher prevalence than the pigs produced locally. The analysis did not indicate the presence of *T*. *asiatica* in the slaughter pigs. The study confirms the endemic nature of *T*. *solium* cysticercosis in Punjab state of India and provides suggestions about reducing the disease burden in pigs and the public.

## Introduction

*Taenia solium* (*T*. *solium*) cysticercosis is an important disease affecting pigs. The pigs become infected after ingesting eggs or gravid proglottis released in the environment after contamination from the faeces of infected human beings. *T*. *solium* cysticercosis is endemic in pigs in India [1] and has been reported from several regions of India [2, 3, 4, 5, 6]. For example, a sero-prevalence of 11.6% of porcine cysticercal antigens was reported from Tamil Nadu, India [7].

Despite recent gains in the understanding of the nature and the prevalence of the disease, and successes in health interventions [8], *T*. *solium* cysticercosis is still endemic and affects mainly poor people in the resource-limited countries [9]. *Taenia solium* human neurocysticercosis has been reported from the human population from many regions of India such as Bihar, Orissa, Uttar Pradesh and Punjab but is rare in Kashmir because of its majority Muslim population [10]. Recently, we demonstrated that human neurocysticercosis-associated active epilepsy results in 2·10 million (95% uncertainty interval 0·99–4·10 million) disability-adjusted life years per annum in India [11].

A thorough inspection of slaughter pigs for *T*. *solium* cysticercosis could help break the life cycle of this parasite. The formal postmortem-inspection at slaughter commonly relies on visual inspection of predilection sites such as heart, diaphragm, masseters, tongue, neck, shoulder, intercostal and abdominal muscles [12]. Exploring other overlooked muscular regions or organs as predilection sites is essential to supplement the current post-mortem inspection procedures. Tongue test was reported to have 70% sensitivity and 100% specificity of in the detection of porcine cysticercosis [13]. Lightowlers et al. (2015) [14] estimated that slicing of the heart, tongue and masticatory muscles at a thickness of approximately 3 mm had a diagnostic sensitivity of approximately 80% in lightly infected animals and recommended tissue dissection as a highly specific and relatively low-cost method for diagnosis of porcine cysticercosis. Compared to tongue examination, ultrasonography has been found to more sensitive (100% versus 91%) but less specific (90% versus 98%), although these differences were not statistically significant [15].

There is very limited information about the prevalence and distribution of *T*. *solium* porcine cysticercosis in the state. A study found the disease prevalence to be 4·23% [2]. However, that study was conducted only in four districts of the state. We are not aware of any study that provided prevalence estimates from other districts or estimated the tissue distribution of the cysts in the state. Further, it is known that scavenging and feral pigs are captured from other states and brought for slaughter and sale in the state. There is anecdotal evidence that these pigs are highly infested with cysts but there is no objective evidence for this. Therefore, the current study aimed to (a) estimate the prevalence of *T*. *solium* porcine cysticercosis by collecting samples from throughout the state, (b) assess the distribution of cysts in pig carcasses, and (c) compare the disease prevalence between pigs reared in the state and those imported from outside the state.

## Methods

### Ethics statement

The Institutional Ethics Committee, Guru Angad Dev Veterinary & Animal Sciences University, Ludhiana did not have any objection to the study as samples were to be collected from slaughtered pigs (Approval number IAEC/2015/97-129).

This study was carried out from August 2016 to July 2017.

### Study area

Punjab is an agrarian state of Northern India (Latitude of 30°4'N and Longitude 75° 5' E) consisting of 22 districts, with a human population of over 27 million [16] and a pig population of more than 32 000 [17]. Most of the pigs are owned by small-holders belonging to low-income groups. As per the official data, 12 240 pigs were slaughtered during the year 2014 [17]. Although no objective data are available, the authors estimate that more than 80% of the pigs are slaughtered in small slaughter shops. The state does not have a pig abattoir except one in the state capital.

### Target and study population

The target population consisted of pigs slaughtered in Punjab, India. The study population consisted of pigs slaughtered in the 44 pig slaughter shops representing all 22 districts (2 per district) of Punjab.

### Sample size

The sample size was calculated using Statulator [18] to be 547 to estimate the prevalence with 95% confidence, a design effect of 1.5 and 2% margin of error and assuming an expected prevalence of 4% based on a previous study [2]. The sample size increased to 730 when a design effect of 2 was applied. We assumed the pig population to be 32000 for these estimates.

The sample size was also calculated to be 393 to detect the disease in the state if present at a prevalence of 2% or above with 95% confidence, assuming the pig population to be 32000 and type I error of 5%, diagnostic sensitivity of 38% and a perfect specificity [19]. Note that this sample size would not be sufficient to demonstrate freedom of disease from each district.

### Sampling

Two slaughter shops were selected in each of the 22 districts of Punjab. A sample of 10–75 pigs slaughtered on the day/s of inspection was post-mortem inspected to identify the presence of *T*. *solium* cysts at each slaughter shop. Pig population and number of pigs inspected for *T*. *solium* cysticercosis in different districts of the state have been described in S1 Table. In addition, pigs imported from outside the state by the slaughter shop owners were selected, if available on the day of sampling. Finally, 40 pigs slaughtered at an abattoir in the capital of the state were also sampled. Although the selection of pigs at the shops/abattoir was not random, we tried to ensure that the pigs are loosely representative of the target population and did not select more than two pigs from a pig owner or a batch (pigs from unknown owners sold by a middleman at the slaughter shop).

### Post mortem inspection

At the time of post-mortem inspection, the masseter and pterygoid muscles, diaphragm, tongue and heart muscles were visually examined, palpated and incised at least twice with long and parallel incisions [2, 20]. The remaining carcass was visually inspected for the presence of cysts. The viable cysts from all infected carcasses were stored in 70% ethanol for the molecular analysis. The viable cysts had cyst walls containing larval cestode with fluid filled bladder and an invaginated scolex [2]. Caseous cysts were considered degenerated cysticercii unless another etiology was evident [2]. The infected pig carcasses were purchased and transported to the laboratory in biohazard bags for further examination.

### Tissue distribution

The distribution of *T*. *solium* cysts in different muscles and organs of the pigs were assessed [21]. The visceral organs were separated, and the remaining carcass was longitudinally cut into two equal parts. The visceral organs along with half the carcass were sliced and the cysts were counted in all the visceral organs and one of the two equally divided half-carcasses. The degenerative and viable cysts were separately counted in the selected muscles and organs. The viable cysts from all the infected carcasses were stored in 70% ethanol for molecular analysis. We explored diaphragm, tongue, hyoid muscle, *Biceps femoris*, *abdominal muscles*, *Serratus Ventralis*, *Longissmus dorsi*, *Intertransversus lumborum*, *Triceps Brachhi*, internal and external masseter muscles. In addition, visceral organs such as spleen, liver, lungs, heart, kidney, brain and oesophagus were also explored. Median cysts per muscle/organ were divided by the respective average muscle/organ weight (gm) to estimate the number of median cysts per gram in the infected muscles/organs.

### DNA extraction

One cyst per carcass was homogenised in a sterile pestle and mortar and 30 mg of the cyst material was used for the extraction. DNA extraction was carried out using HiPurA mammalian genomic purification spin kit (Himedia) as per manufacturer’s instructions. The eluted DNA was stored at—20°C till further use.

### Polymerase chain reaction

The published oligonucleotide primer sequences were used for the PCR amplification (Table 1) and were synthesized by Eurofins Pvt. Ltd.

**Table 1:** Oligonucleotide primers used for detection of *T*. *solium* cysts from naturally infected pigs in Punjab, India.

Sr No	Target gene	Primer name	Primer Sequence
1	Large subunit rRNA gene	TBR-3	(5′-GGC TTG TTT GAATGG TTT GAC G-3′)
		TBR-6	(5′-GCT ACT ACA CCT AAA TTC TAA CC-3′)
2	Cytochrome oxidase I gene	JB3	(5′-TTTTTTGGGCATCCTGAGGTTTAT-3′)
		JB4.5	(5′-TAAAGAAAGAACATAATGAAAATG-3′)
3	Internal transcribed spacer 1 gene	NAP 9	(5′-AACAGGTCTGTGATGCCCT-3′)
		4S	(5′-CTAGATGCGTTCGAA(G/A)TGTCGATG-3′)
4	Diagnostic antigen gene, Ts14	gTs14F	(5′-ATGCGTGCCTACATTGTGCTTCTC-3′)
		gTs14-R2	(5′-GCAGTTTTTTTCTTAGGACCTTTGCAGTG-3′)

The PCR reaction was carried out in a total reaction volume of 25μl containing 12·5μl master mix (GoTaq green Promega), 1μl each of forward and reverse primers (10 pmol), 5·5μl nuclease free water and 5μl DNA template (20–100 ng). The cycling condition for uniplex PCR was pre-denaturation and polymerase activation step at 94°C for 2 min, with 35 amplification cycles (denaturation at 94°C for 30 sec, annealing for large subunit rRNA gene and internal transcribed spacer 1 gene at 60°C, ITS1 gene at 56°C and cytochrome oxidase I gene at 50°C for 30 sec and elongation at 72°C for 1 min) and a final elongation step at 72°C for 5 min in Master cycler Pro (Eppendorf, T-Gradient) thermal cycler. The morphologically confirmed *T*. *solium* cysts were used as positive control(s). The DNA extraction control and no template controls were used as negative controls. The 10μl of amplified product was subjected to the 1.5% agaorse gel electrophoresis and gel documentation.

### Sequencing and phylogenetic analysis

Due to suitability of the mitochondrial genes to compare polymorphisms in *T*. *solium* and to establish phylogenetic trees for related *Taenia* species (*T*. *solium*, *T*. *saginata* and *T*. *asiatica)*, the positive amplicons using JB3 and JB4.5 primers against mitochondrial cytochrome oxidase 1 gene were purified and sequenced. DNA sequencing was performed in both directions by AgriGenome, Kerala, India. Sequence chromatograms were analysed using the Bioedit, ClustalW and Mega 6·0 computer software programmes. Sequences were matched using NCBI BLAST software, aligned and compared with previously published sequences of *T*. *solium* (Gene Bank accession numbers AF360870·1, FM958310·1, GU097653·1, EF076752·1, FN995658·1, and FN995666·1) using Mega 6.0 computer software. The sequences were also compared with *T*. *saginata* (Gene Bank accession number AB533172·1) and *T*. *asiatica* (Gene Bank accession number AB107236·1). *Echinococcus granulosus* (Gene Bank accession number FJ608752.1) and *E*. *multilocularis* (Gene Bank accession number AB461420·1) were used as an out group. Distance-based analysis was conducted and a tree was constructed using the neighbour-joining algorithm and Mega 6·0 software.

### Statistical analysis

The apparent and estimated true prevalence of porcine cysticercosis was estimated with 95% confidence interval (CI) using Epi Tools [22, 23]. Univariable logistic regression analyses were performed to assess the effect of age, sex and pig rearing status on the disease prevalence using a binary outcome variable (cysts present: 1/0).

For tissue distribution, the median number of *T*. *solium* cysts from all the positive pigs were estimated from the total number of cysts counted from muscles and visceral organs of all the 19 pig carcasses. The Wilcoxon rank sum test was used to compare the number of cysts present in male and female pigs as the distribution of cyst counts was right skewed and therefore, the assumptions of the parametric 2-sample t-test were invalid. However, the difference in viable and degenerative cysts within a carcass was approximately normally distributed, and therefore, the paired t-test was used to test if the mean difference was significantly different from zero. Fisher’s exact test (one tailed) was conducted to compare the disease prevalence between formal abattoir and slaughter shop inspected pigs from Punjab. All the analyses were conducted in R-statistical program unless indicated otherwise (R statistical package version 3.4.0, R Development Core Team (2015), http://www.r-project.org).

## Results

### Prevalence of porcine cysticercosis

We contacted 71 slaughter shop owners of which 44 consented to participate in the study (response rate = 61%). We inspected 642 pigs reared in Punjab, of which 9 had *T*. *solium* cysts with an apparent prevalence of 1·40% (95% CI: 0·74%, 2·64%) and the estimated true prevalence of 3·69% (95% CI: 1·95%, 6·95%). Pigs imported from outside the state (n = 450) were selected from four districts, namely Ludhiana (138), Jalandhar (256), Firozpur (21) and Patiala (35), of which 15 were positive indicating an apparent prevalence of 3·33% (95% CI: 2·03%, 5·43%) and the estimated true prevalence of 8·77% (95% CI: 5·34%-14·28%). Pigs imported from outside had a significantly higher prevalence (odds ratio: 2·58; 95% CI: 1·12, 5·98; p-value: 0·026) than the pigs reared in the state. None of samples was positive from the pigs sampled from the abattoir in the state capital. Data analysis revealed high disease prevalence from the slaughter shops as compared to the formal abattoir, although the association was not statistically significant (p-value = 0·58).

Detailed information on the district-wise apparent and estimated true prevalence (95% CI) is presented in Table 2. The disease was recorded only from four districts of the state.

**Table 2:** Apparent and estimated true prevalence for *T*. *solium* cysticercosis in pigs reared in different districts in Punjab, India.

District	Number of pigs inspected	Number of pigs positive	Apparent prevalence	95% CI	Estimated true prevalence	95% CI
**Pigs reared within the state**						
Jalandhar	143	5	3· 5	1·5–7·9	9·2	3·9–20·8
Ludhiana	60	2	3·3	0·9–11·4	8·8	2·4–29·9
Firozpur	24	1	4·2	0·7–20·2	11·0	0·6–53·2
Patiala	55	1	1·8	0·3–9·6	4·8	0·2–25·2
Other districts^a^	360	0	0·0^b^	0·0–1·0	0·0	0·0–2·8
*Punjab*	*642*	*9*	*1·4*	*0·7–2·6*	*3·7*	*1·9–6·9*
*Chandigarh*^a^ *(Formal abattoir)*	*40*	*0*	*0·0* ^b^	*0·0–8·8*	*0·0*	*0·0–23·0*
**Pigs imported from other states**						
Jalandhar	256	12	4·7	2·7–8·0	12·3	7·1–21·1
Ludhiana	138	3	2·2	0·7–6·2	5·7	1·9–16·3
Ferozepur and Patiala	56	0	0·0 ^b^	0·0–6·4	0·0	0·0–16·9
*Total*	*450*	*15*	*3·3*	*2·0–5·4*	*8·8*	*5·3–14·3*

^a^None of the sample was found positive in the districts Gurdaspur, Amritsar, Tarn Taran, Kapurthala, Shaheed Bhagat Singh Nagar, Hoshiarpur, Rupnagar, Sahibzada Ajit Singh Nagar, Faridkot, Moga, Muktsar, Bathinda, Mansa, Fatehgarh sahib, Sangrur, Barnala, Fazilka, Pathankot and state capital Chandigarh

^b^Inestimable

### Polymerase chain reaction

The polymerase chain reaction confirmed the *T*. *solium* cysts and revealed the amplicon sizes of 286 bp, 420 bp, 1150 bp and 333 bp from all the 24 positive samples targeting large subunit rRNA gene, cytochrome oxidase I gene, internal transcribed spacer 1 gene and the diagnostic antigen Ts14 gene, respectively. The PCR did not show any reaction with the negative controls.

### Sequencing and phenogram construction

**Fig 1:**
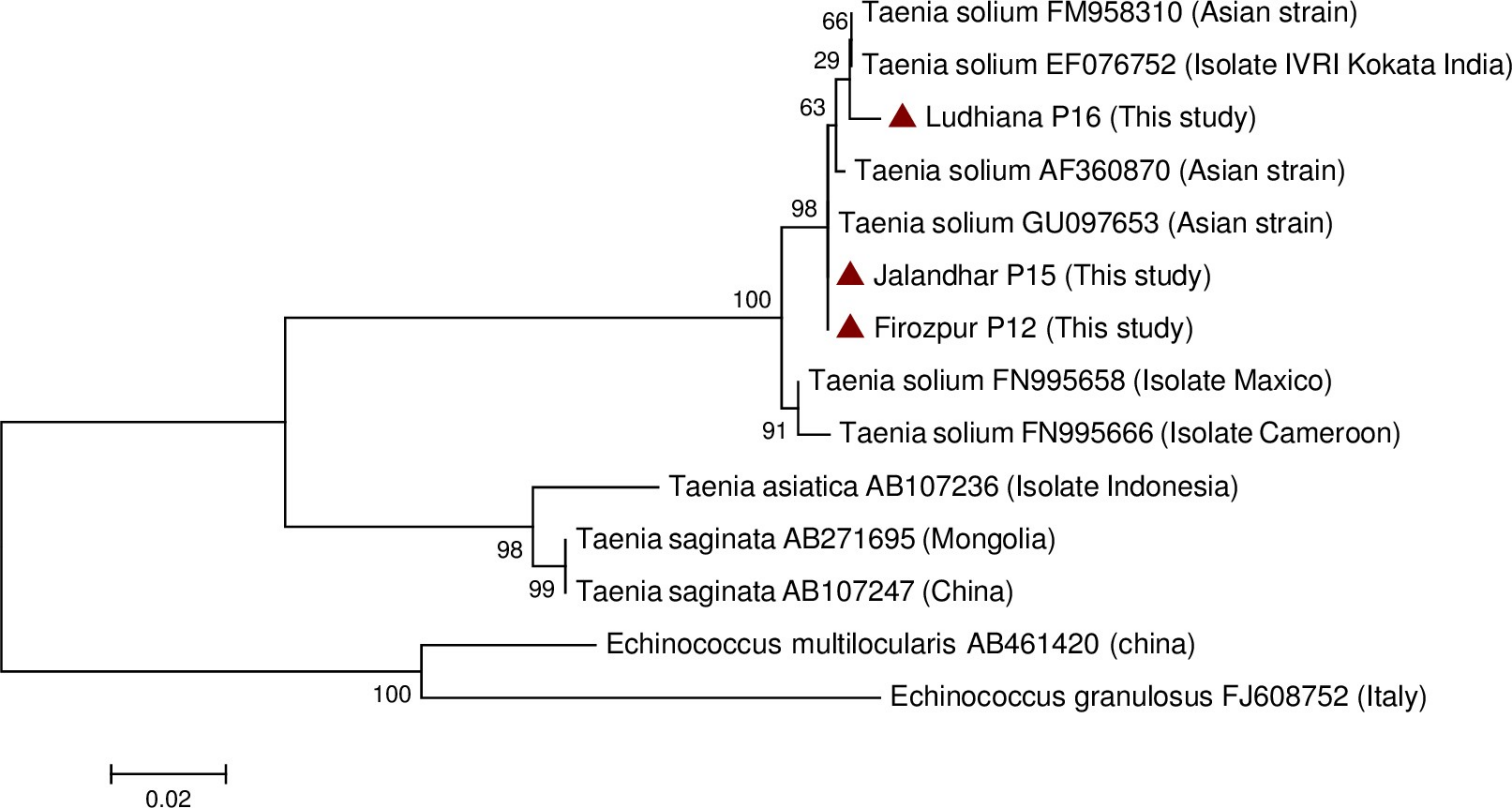
Phenogram construction of the cytochrome oxidase 1 gene of *Taenia solium* isolates from naturally infected pigs in Punjab (India) along with reference strains.

The phylogenetic tree based on the alignment of partial cytochrome oxidase 1 sequences indicated that all positive samples were found to be clustered with the *T*. *solium* Asian genotype (Fig 1).

The analysis did not indicate the presence of *T*. *asiatica* in the inspected pigs. The three cytochrome oxidase 1 gene *T*. *solium* partial coding sequences had an alignment score of 99% among themselves and 95–99% with the previously reported [2] *T*. *solium* sequences from Punjab. The sequence identity of all the sequences from this study was in the range of 97–99% with the published *T*. *solium* sequences in the database having accession numbers AF360870.1, FM958310.1, GU097653.1, EF076752.1, FN995658.1, and FN995666.1. All the three isolates from this study showed 88% similarity with the sequences of *T*. *sagianta* from China (AB107247) and Mongolia (AB271695) whereas as no significant similarity was found with *Taenia asiatica* (AB107236).

### Risk factor evaluation

The explanatory variable ‘pig rearing area’ was significantly associated with the outcome variable (OR 3·0; 95% CI 1·12, 5·98; p < 0·01). The prevalence was not significantly different in different age (OR 1·2; 95% CI 0·5, 3·3; p = 0·7) or sex (OR 0·6; 95% CI 0·3, 1·5; p-value = 0·3) groups. Multivariable analyses were not conducted as only one explanatory variable was significant in univariable analyses.

### Tissue distribution

Of the 24 positive pig carcasses, 19 were purchased and further examined in the laboratory. The carcasses weighed 40.7 kg on the average (range 36–50 kg) and contained a median of 897 (range 526–1964) cysts per infected pig carcass. Of these, a median of 280 cysts (range 160–720) were viable and 575 (range 350–1244) were degenerative. The mean cyst count was significantly different between degenerative and viable cysts (291; 95% CI 231–351) (p-value: < 0.001). However, the median number of cysts was not significantly different between male (947) and female (815) pigs.

Maximum median numbers of cysts were counted in *triceps brachi* muscle (median 99; range 48–782 cysts), *serratus ventralis* (93; 21–334) followed by *inter transversus muscle* (median 89; 45–231) and the external *masseter* muscle (median 63; 36–194). The cyst burden was highly variable between organs and muscle groups. High loads were observed in the masseter, forelimb(s) and hind limb(s) muscles whereas the low counts were observed in the tongue, oesophagus, brain, heart and hyoid muscles. The median numbers of cysts in all the positive carcasses has been presented in Table 3.

**Table 3:** Number of *T*. *solium* cysts recorded in different muscles and organs in naturally infected pigs in Punjab, India.

Muscle/organ^a^	Median	Minimum	Maximum	Median count (per 10 gram)
Abdominal muscles	44	6	330	1
Biceps femoris	72	21	231	1
Brain	24	7	42	3
Diaphragm	55	20	172	3
External masseter	63	36	194	12
Heart	31	15	85	2
Hyoid muscle	21	6	54	3
Internal masseter	62	36	127	27
Intertransversus lumborum	89	45	231	2
Longissmus dorsi	42	10	120	1
Oesophagus	10	2	19	2
Serratus ventralis	93	21	234	3
Tongue	36	0	78	4
Triceps brachii	99	48	782	2

^a^No cysts were recorded from spleen, liver, lungs and kidneys

## Discussion

The current study re-confirms that *T*. *solium* cysticercosis is endemic in pigs in the Punjab state of India. We estimated a true prevalence of 3·7% (95% CI: 1·95%, 6·95%) in pigs reared in the state. A previous study also reported an apparent prevalence of 4·23% (95% CI: 2·8, 6·3) from the selected areas in the state [2]. Higher apparent prevalence of 5·14% has been reported from Uttar Pradesh [24] and 9.5% from the Assam state of India [5]. Many factors such as geographic area, rearing practices and pig owners’ hygienic practices play important role in the occurrence of porcine cysticercosis [25]. Therefore, the geographical distribution of infection varies in different states of India [26]. The endemic nature of the parasite demands implementation of control measures in the affected areas.

The prevalence estimate in this study is likely to be an under-estimate of the actual prevalence. A low response rate (61%) could have caused a selection bias as only the shop owners receiving relatively healthier pigs might have agreed to participate in the study. Additionally, only two shops per district were selected. This might have led to an underestimation of disease prevalence as it is possible that those butchers who were aware of a high rate of infection among their carcasses were also more likely to have refused permission for the study for fear of the legal consequences or fear of loss of income. Further, the pigs were inspected with the inspection procedure reported to have a diagnostic sensitivity and specificity of 38% and 100%, respectively [21]. Although false positive detections were unlikely, it is quite likely that we could have missed a number of positive carcasses and only detected heavily infected pigs, further leading to an under-estimation of the disease prevalence. Slicing of the heart, tongue and masticatory muscles at a thickness of approximately 3 mm has been reported to have a diagnostic sensitivity of approximately 80% in lightly infected animals and therefore this method is recommended to be used in the future research [14]. Availability of a highly specific and sensitive serologic test could also overcome this limitation in the future. The use of battery of tests such as a combination of both serological and post-mortem inspection could improve diagnostic sensitivity in the future research.

The disease was detected from four of the 22 districts in the state. We calculated sample size for estimating the disease prevalence in the state and to detect the disease if it is present at more than 2% level. However, the sample size was not sufficient to detect the disease in districts. Therefore, we cannot be sure that the districts from where the carcasses were found to be negative for cysticercosis do not have the disease. Further studies by calculating sample size sufficient to detect the disease at a district or sub-district level should be conducted to detect the disease or to demonstrate freedom from the disease in these districts.

The selection of slaughter shops from within districts was not random due to a lack of a database of the numbers and types of pig slaughter shops and an absence of animal identification systems in the state. This could have biased our results but for the first time we selected samples from all districts of the state and tried to ensure representative selection of pig carcasses at pig shops. Further, some slaughter shop owners declined to sell infected pig carcasses for the study due to fear of any legal action which contributed to the low number of carcasses in the tissue distribution study (only 19 out of 24 infected whole carcasses could be inspected to assess tissue distribution).

Interestingly, none of the pigs from the formal abattoir was found to be positive. This could be due to the reason that only healthier pigs are submitted for slaughter to formal abattoirs. This suggests that meat inspection in formal abattoirs alone is not sufficient to control the disease. Therefore, inspection should also be conducted in unorganised slaughter shops.

Pigs imported from outside the state had significantly higher prevalence than those reared in the state suggesting that they are a greater risk for the spread of *T*. *solium* cysticercosis in the state. The anecdotal information received from the slaughter shop owners revealed that most of these pigs are scavenging or feral pigs brought from other states and sold at discounted rates. Scavenging of pigs has been reported to be an important risk for porcine cysticercosis [2]. Similarly, feral pigs have been reported to be an important source for infectious diseases [27]. We recommend that all pigs imported from outside the state must be inspected for *T*. *solium* cysticercosis during slaughter in the state. Additionally, an import restriction such as tongue inspection or serological testing of all these pigs at the port of entry is also recommended.

We detected high cyst loads in the infected pig carcasses. High cyst load of 76–80340 cysts in pig carcasses has also been reported from Tanzania [28]. Similarly, approximately 20% of the carcasses contained more than 200 cysts and 93% of the infected carcasses had viable cysts in an endemic focus in Nepal [29]. This indicates that high cyst loads are not uncommon in the endemic areas. Presence of heavy cyst load in infected carcasses undoubtedly raises a public health concern as it increases the number and risk of infected pork servings consumed by the pork consumers in the state.

The cysticerci were recovered from all important muscles such as diaphragm, tongue, hyoid muscle, biceps femoris, abdominal muscles, serratus ventralis, longissmus dorsi, intertransversus lumborum, triceps brachii, external masseter and internal masseter muscles. The cysts were also recorded from heart, brain, and oesophagus, whereas no cyst was recovered from the kidneys, liver, spleen and lungs. Similar observations have also been recorded in Tanzania and Nigeria [28, 30].

Phylogenetic analysis revealed the presence of Asian genotypes of *T*. *solium* from all of the samples. Molecular detection and phylogenetic analysis were performed on one cyst per infected pig. Therefore, the presence of species other than *T*. *solium* could not be ruled out. However, none of the pigs examined to determine the tissue distribution had *Taenia* cysts in their livers, suggesting the absence of *T*. *asiatica* in the infected pigs. The Asian genotype of *T*. *solium* has been reported from many other Asian countries, including China, Thailand and Indonesia [31]. Many genotyping studies have targeted the mitochondrial cytochrome oxidase 1 gene and have reported African/Latin American genotypes of *T*. *solium* from East African countries [32]. *Taenia asiatica* and *T*. *solium* (African/Latin American genotype) were not detected in the current study.

The choice of diagnostic test for the diagnosis of *T*. *solium* cysticercosis in pigs is an important issue. We performed post-mortem inspection of slaughter pigs in the current study. The carcass examination is a highly specific method and can readily differentiate between viable and degenerative cysts. However, it reduces carcass quality and the diagnostic sensitivity of carcass examination is low barring the whole carcass examinations. A serological test could reduce labour, costs involved and maintain carcass quality but most of the available serological tests also show a lower sensitivity in rural pigs having a low cyst burden [20, 33, 34]. In the absence of reliable serological test, we believe our choice of post-mortem inspection to be appropriate in the current situation.

Many risk factors such as scavenging of pigs, deworming and vaccination status of pigs, presence or absence of latrines in the households, farmer’s level of education, household size, annual family income, habit of consuming of raw pork and hand-washing after using the toilet, and human taeniosis status of a pig farmer that may be associated with the infection in pigs could not be evaluated due to the lack of information of farm level factors in this study. Therefore, further studies involving farms should be conducted to investigate farm level risk factors.

The study found an estimated true prevalence of 3.69% in domestic and 8.77% in imported pigs slaughtered in the Punjab state of India. Many factors such as underestimation of the prevalence due to missing of mildly-infected carcasses, low participation and lack of representative sampling, and a low sample size to molecularly differentiate *Taenia* species undermined the outcome of this study. However, absence of cysts in liver of the infected pigs and presence of heavily infected carcasses containing viable cysts indicates that *T*. *solium* cysticercosis is an important food safety concern in Punjab and should be tackled as a priority.

Pigs slaughtered at slaughter shops should be regularly examined to ensure that the pork is safe for human consumption. Special attention should be paid to the scavenging and feral pigs imported for slaughter and sale in the state. A disease control policy should be developed and implemented using a One Health approach to control the disease both in pigs and the public.

## Supporting information

S1 Table Detailed information on the total pigs and number of pigs inspected for *T*. *solium* cysticercosis in different districts in Punjab, India. (DOCX)
