# Supplementary material for: Prevalence and distribution of Taenia solium cysticercosis in naturally infected pigs in Punjab, India
Source: PLoS Negl Trop Dis. 2018 Nov 15;12(11):e0006960. doi: 10.1371/journal.pntd.0006960 (PMC6264866; doi:10.1371/journal.pntd.0006960)
Supplement: S1 Table — (DOCX) [file pntd.0006960.s001.docx]

| **District** | **Total number of pigs (BAHS, 2014)^a^** | **Number of pigs inspected** |
| --- | --- | --- |
| Gurdaspur | 934 | 20 |
| Amritsar | 690 | 20 |
| Tarn Taran | 1123 | 20 |
| Kapurthala | 356 | 20 |
| Jalandhar | 1430 | 143 |
| Nawan Shahar | 243 | 20 |
| Hoshiarpur | 532 | 20 |
| Ropar | 1290 | 20 |
| Mohali | 3590 | 20 |
| Ludhiana | 8064 | 60 |
| Firozpur | 2007 | 24 |
| Faridkot | 295 | 20 |
| Moga | 984 | 20 |
| Muktsar | 745 | 20 |
| Bathinda | 416 | 20 |
| Mansa | 1459 | 20 |
| Patiala | 3241 | 55 |
| Fatehgarh Sahib | 2374 | 20 |
| Sangrur | 1696 | 20 |
| Barnala | 752 | 20 |
| Fazilka & Pathankot | 779 | 40 |
| Punjab | 32221 | 642 |
| Chandigarh (UT)^b^ | 135 | 40 |

^a^Basic Animal Husbandry Statistics

^b^Union Territory
